# Home-Based Exercise Program Ameliorates Renal Function Decline in Patients With CKD Stage 4

**DOI:** 10.1016/j.ekir.2022.01.006

**Published:** 2022-01-07

**Authors:** Keika Adachi, Kiyotaka Uchiyama, Kaori Muraoka, Takashin Nakayama, Marie Yasuda, Kazutoshi Miyashita, Hirobumi Tokuyama, Shu Wakino, Hiroshi Itoh

**Affiliations:** 1 Department of Endocrinology, Metabolism and Nephrology, Keio University School of Medicine, Tokyo, Japan; 2 Department of Rehabilitation Medicine, Kitasato University Kitasato Institute Hospital, Tokyo, Japan; 3 Department of Nephrology, Tokyo Dental College Ichikawa General Hospital, Chiba, Japan; 4 Department of Nephrology, Tokushima University, Tokushima, Japan

**Keywords:** chronic kidney disease, estimated glomerular filtration rate, home-based exercise, randomized controlled trial, renal function

Aerobic and resistance training in patients with chronic kidney disease (CKD), including those with predialysis CKD and those with hemodialysis or peritoneal dialysis, is increasingly known to be beneficial.^1^^,^^2^ With respect to the effects of exercise on renal function, randomized controlled trials (RCTs) revealed that exercise intervention significantly resulted to estimated glomerular filtration rate (eGFR) decline.^3^^,^^4^ In a meta-analysis of 7 RCTs, a significant improvement in eGFR of +2.22 ([0.68–3.76] ml/min per 1.73 m^2^) was observed on exercise intervention for 12 to 24 weeks.^1^ Nevertheless, these effects on renal function were inconclusive because of the limited number of subjects and variation in the intervention methods and subject characteristics. In particular, most RCTs included patients with diabetes, obesity, and mild renal impairment mainly with stage 3 CKD (eGFR of 30–60 ml/min per 1.73 m^2^).

We recently revealed that a home-based exercise program including aerobic exercise and resistance exercise improved aerobic capacity as evaluated by the incremental shuttle walking test, health-related quality of life, serum C-reactive protein (an inflammatory marker), and acylcarnitine-to-free carnitine ratio in patients with stage 4 CKD (eGFR of 15–30 ml/min per 1.73 m^2^).^5^ In addition, the exercise intervention decreased urinary excretion of liver-type fatty acid-binding protein as a biomarker of CKD progression, suggesting possible beneficial effects on kidney function. Although the renal function change evaluated with combined urea and creatinine clearance was not significantly different between the control and exercise groups, the difference in the baseline rate of CKD progression was not considered.

Therefore, we performed a *post hoc* analysis of our previous RCT to primarily clarify the effect of the exercise intervention on the change in eGFR slope from the pre-exercise to midexercise period. Furthermore, to clarify the lasting effect of exercise, we also evaluated the eGFR slope postexercise.

## Results

### Patients and Baseline Characteristics

Among 242 outpatients with stage 4 CKD, 46 patients were randomly assigned to the control group (*n =* 23) and the exercise group (*n =* 23) in the previous RCT, and all of these patients were included in this *post hoc* analysis (Supplementary Figure S2).

The clinical characteristics of study participants at baseline (at the start of exercise intervention), including eGFR slope, changes (Δ) in body weight with body mass index, blood pressure (BP), and heart rate during the pre-exercise period, are summarized in Table 1. eGFR measurements for eGFR slope calculation were performed 7 (6–9), 4 (4–5), and 4 (3–5) times during the pre-exercise, midexercise, and postexercise periods, respectively. The control and exercise groups had no significant differences in these clinical parameters. Moreover, baseline physical activity evaluated using the International Physical Activity Questionnaire did not significantly differ between the groups (26.5 ± 25.8 vs. 22.8 ± 17.7, *P* = 0.57). In addition, eGFR, body weight with body mass index, BP, and heart rate 48 weeks before the intervention, at the start of intervention, at the end of the intervention, and 24 weeks after the end of intervention were compared between the groups (Supplementary Table S1).

**Table 1:** Demographic, clinical, and biochemical data of the study groups

Variables	All (N = 46)	Control (*n =* 23)	Exercise (*n =* 23)	*P* value
Age (yr)	73 (69–78)	76 (69–78)	72 (69–79)	0.74
Male/female, *n* (%)	33/13 (72/28)	16/7 (70/30)	17/6 (74/26)	1
Diabetes, *n* (%)	14 (30)	7 (30)	7 (30)	1
CCVD, *n* (%)	12 (26)	5 (22)	7 (30)	0.74
Smoking, *n* (%)	21 (46)	10 (44)	11 (48)	1
Kidney function				
Renal CrCl (ml/min per 1.73 m^2^)	32.6 ± 10.0	32.2 ± 8.1	34.8 ± 10.5	0.35
Renal urea Cl (ml/min per 1.73 m^2^)	14.8 ± 4.7	14.7 ± 4.1	16.5 ± 5.3	0.21
Average Cr and urea Cl (ml/min per 1.73 m^2^)	23.7 ± 7.1	23.5 ± 5.8	25.7 ± 7.5	0.28
Urine protein (g/d)	0.6 (0.2–2.3)	0.6 (0.3–1.4)	0.5 (0.2–3.4)	0.8
Urine albumin (mg/d)	404.8 (77.9–1513.6)	404.8 (82.6–948.5)	390.0 (84.7–2151.0)	0.57
Urine L-FABP (μg/d)	16.9 (5.7–47.5)	16.8 (7.9–31.2)	17.1 (4.4–67.2)	0.42
eGFR (ml/min per 1.73 m^2^)	23.1 ± 4.8	23.8 ± 4.5	22.4 ± 5.1	0.32
BMI (kg/m^2^)	23.9 ± 4.5	23.0 ± 4.3	24.7 ± 4.6	0.23
GNRI	100.7 ± 9.8	99.3 ± 9.3	102.2 ± 10.3	0.32
nPCR (g/kg/d)	0.93 ± 0.20	0.88 ± 0.17	0.99 ± 0.21	0.06
Hemoglobin (g/dl)	12.0 ± 1.6	11.8 ± 1.2	12.3 ± 1.9	0.33
Hemoglobin A1c (%)	6.1 ± 0.7	6.1 ± 0.8	6.0 ± 0.6	0.84
Glycated albumin (mmol/l)	15.4 ± 2.7	16.0 ± 2.9	14.8 ± 2.4	0.14
Fasting blood sugar (mg/dl)	116.2 ± 21.6	115.1 ± 17.4	17.3 ± 25.5	0.73
HOMA-IR	3.0 (1.5–4.8)	2.9 (1.6–5.4)	3.1 (1.6–4.6)	0.78
Albumin (g/dl)	3.7 ± 0.4	3.7 ± 0.3	3.7 ± 0.6	0.92
Calcium (mg/dl)	9.2 ± 0.3	9.3 ± 0.3	9.2 ± 0.4	0.65
Phosphorus (mg/dl)	3.6 ± 0.5	3.6 ± 0.6	3.7 ± 0.5	0.62
PTH (pmol/l)	106.8 ± 65.2	98.6 ± 57.3	115.1 ± 72.6	0.4
CRP (mg/dl)	0.05 (0.03–0.16)	0.12 (0.03–0.35)	0.05 (0.03–0.11)	0.11
IL-6 (pg/ml)	2.5 (1.8–4.5)	2.6 (2.1–4.6)	2.3 (1.5–4.2)	0.21
Total cholesterol (mg/dl)	197.2 ± 37.1	194.6 ± 38.8	199.7 ± 36.0	0.64
LDL cholesterol (mg/dl)	108.0 ± 28.9	109.3 ± 30.6	106.7 ± 27.6	0.76
HDL cholesterol (mg/dl)	50.9 ± 14.4	49.8 ± 16.5	51.9 ± 12.2	0.63
Triglyceride (mg/dl)	141.5 ± 52.8	136.4 ± 51.2	146.7 ± 55.1	0.52
BNP (pg/ml)	47.7 (26.3–82.2)	47.5 (29.6–64.2)	47.9 (23.5–84.5)	0.56
hANP (pg/ml)	47.7 (27.0–68.9)	50.1 (27.1–64.7)	47.0 (26.5–68.5)	0.88
Free carnitine (μmol/l)	50.0 ± 10.8	48.1 ± 10.9	51.9 ± 10.6	0.24
Acylcarnitine (μmol/l)	17.2 ± 5.1	16.1 ± 4.6	18.4 ± 5.3	0.13
AC/FC	0.35 ± 0.12	0.34 ± 0.11	0.36 ± 0.13	0.56
Exercise capacity				
ISWT (m)	367.6 ± 136.2	363.9 ± 131.6	371.3 ± 143.5	0.86
Handgrip strength (kg)	27.7 ± 8.4	25.3 ± 7.7	30.0 ± 8.6	0.06
Quadriceps strength (kg)	27.2 ± 11.5	25.2 ± 9.9	29.3 ± 12.8	0.23
IPAQ	24.7 ± 22.0	26.5 ± 25.8	22.8 ± 17.7	0.57

AC/FC, acylcarnitine-to-free carnitine ratio; BMI, body mass index; BNP, brain natriuretic peptide; CCVD, cerebrovascular/cardiovascular disease; Cl, clearance; Cr, creatinine; CrCl, creatinine clearance; CRP, C-reactive protein; eGFR, estimated glomerular filtration rate; GNRI, geriatric nutritional risk index; hANP, human atrial natriuretic peptide; HDL, high-density lipoprotein; HOMA-IR, homeostasis model assessment of insulin resistance; IL-6, interleukin-6; IPAQ, International Physical Activity Questionnaire; ISWT, incremental shuttle walking test; LDL, low-density lipoprotein; L-FABP, liver-type fatty acid-binding protein; nPCR, normalized protein catabolism rate; PTH, parathyroid hormone.

### Effect of the Home-Based Exercise Program on eGFR Slope

Linear mixed models confirmed that the eGFR slope with the estimated marginal mean in the exercise group changed from −2.19 ± 0.79 ml/min per 1.73 m^2^/yr during the pre-exercise period to −1.60 ± 0.79 ml/min per 1.73 m^2^/yr midexercise and −2.19 ± 0.79 ml/min per 1.73 m^2^/yr during postexercise, whereas −1.55 ± 0.79, −4.36 ± 0.79, and −3.94 ± 0.79 ml/min per 1.73 m^2^/yr were observed in the control group, respectively (Table 2 and Supplementary Figure S3). In contrast to the control group, the exercise group had a 3.40 ml/min per 1.73 m^2^/yr (95% CI, 0.65 to 6.15; *P* = 0.02) mean change in eGFR slope during the exercise period, but the difference diminished during the postexercise period (2.39 ml/min per 1.73 m^2^/yr [95% CI, −0.36 to 5.14; *P* = 0.09]).

**Table 2:** Linear mixed-effects model of the exercise intervention on outcomes

Variables	Control group^a^			Exercise group^a^			Effect of intervention^b^			
	Pre-exercise	Midexercise	Postexercise	Pre-exercise	Midexercise	Postexercise	ΔMid−pre-exercise	*P* value	ΔPost−pre-exercise	*P* value
eGFR slope (ml/min per 1.73 m^2^/yr)	−1.55 ± 0.79	−4.36 ± 0.79	−3.94 ± 0.79	−2.19 ± 0.79	−1.60 ± 0.79	−2.19 ± 0.79	3.40 ± 1.38	0.02	2.39 ± 1.38	0.09
ΔBody weight (kg)	0.62 ± 0.74	0.90 ± 0.74	−0.38 ± 0.74	0.38 ± 0.74	0.11 ± 0.74	−0.50 ± 0.74	−0.55 ± 1.75	0.75	0.12 ± 1.75	0.95
ΔBody mass index (kg/m^2^)	0.23 ± 0.28	0.36 ± 0.28	−0.15 ± 0.28	0.14 ± 0.28	0.11 ± 0.28	−0.22 ± 0.28	−0.16 ± 0.65	0.81	0.015 ± 0.65	0.98
ΔSystolic blood pressure (mm Hg)	1.91 ± 4.35	2.60 ± 4.35	4.65 ± 4.35	0.63 ± 4.36	1.80 ± 4.36	−2.37 ± 4.36	0.48 ± 10.25	0.96	−5.74 ± 10.25	0.58
ΔDiastolic blood pressure (mm Hg)	0.95 ± 2.61	1.00 ± 2.61	−0.44 ± 2.61	−0.18 ± 2.62	0.17 ± 2.62	−2.62 ± 2.62	0.30 ± 6.09	0.96	−1.04 ± 6.09	0.86
ΔMean blood pressure (mm Hg)	1.25 ± 2.99	1.51 ± 2.99	1.23 ± 2.99	0.090 ± 2.99	0.71 ± 2.99	−2.53 ± 2.99	0.36 ± 7.01	0.96	−2.61 ± 7.01	0.71
ΔHeart rate (bpm)	4.47 ± 2.97	−2.18 ± 2.97	1.12 ± 2.97	3.93 ± 2.98	−7.21 ± 2.98	5.01 ± 2.98	−4.48 ± 6.94	0.52	4.36 ± 6.94	0.53

bpm, beat per minute; eGFR, estimated glomerular filtration rate.

a Estimated marginal mean and SE.

b Effect of interaction term (time × group) with SE.

### Effect of the Home-Based Exercise Program on Secondary Outcomes

Δbody weight with Δbody mass index, Δsystolic BP, Δdiastolic BP, Δmean BP, and Δheart rate did not change significantly during the exercise period (*P* = 0.51, 0.56, 0.97, 0.62, 0.75, and 0.38, respectively) or during the postexercise period (*P* = 0.66, 0.65, 0.78, 0.95, 0.87, and 0.41, respectively) between the control and exercise groups (Table 2).

## Discussion

Initially, we revealed that 24 weeks of home-based training slows the decrease in eGFR among patients with stage 4 CKD from the pre-exercise to midexercise period, whereas this effect deteriorated in the postexercise period.

Similar to our results, the previous RCT including patients with stages 3 to 4 CKD revealed a significant mean difference in the rate of change of eGFR calculated from serum creatinine between the usual care and rehabilitation groups, with the rehabilitation group having a slower decline.^3^ In addition, a meta-analysis of 7 RCTs, among which 3 were from the United States, whereas the rest were from the United Kingdom, Denmark, China, and Brazil, suggested that exercise intervention for 12 to 24 weeks might improve eGFR.^1^ Nevertheless, the benefit of exercise on renal outcomes in patients with advanced CKD remained to be clarified because these RCTs were designed to include patients with diabetes, obesity, and mild renal impairment with mainly stage 3 CKD. Our results suggest that the beneficial effect of exercise intervention on renal outcomes was reproduced in patients with stage 4 CKD, regardless of the complications of diabetes and obesity, combined with the finding of our previous RCT revealing an improvement in liver-type fatty acid-binding protein, a biomarker of CKD progression,^6^ in the exercise group.^5^ Unfortunately, we could not evaluate the mechanism underlying the beneficial effect of exercise on the rate of eGFR decrease, whereas the improvement in kidney function has often been attributed to the improvement in vascular health or improvement in endothelial dysfunction.^3^^,^^7^^,^^8^

Johnson *et al.*^9^ reported a lasting or “legacy” effect of exercise on the maintenance of peak oxygen uptake in 10 years, although to our knowledge, there have been no studies to evaluate the “legacy” effect of exercise intervention on renal function. Similarly, we could not find a significant improvement in eGFR slope from the pre-exercise to postexercise period. We did not restrict the home-based exercise after the end of the intervention in the exercise group, such as what Johnson *et al.*^9^ did; actually, it is unrealistic and unethical to instruct participants to stop exercising. Therefore, it is also likely that acquired exercise habits from the 24-week intervention period in the exercise group led to the persistence of beneficial effects in that group during the postexercise period. To evaluate the effect of habitual exercise separately from this “legacy” effect, additional long-term trials with evaluation of physical activities after the end of intervention are necessary.

There are several limitations in this study. First, all subjects in the control group and the exercise group were given exercise training after the intervention period without monitoring their adherence, and the amount of exercise during the postexercise period depended on the patients’ autonomy; therefore, the amount of exercise might be unbalanced between the control and exercise groups. Second, the sample size was too small and the trial period was too short to evaluate the effect of the exercise program on hard outcomes, including the initiation of renal replacement therapy, cardiovascular outcomes, and death. Future trials with a larger number of participants and longer follow-up durations are warranted to evaluate the effects of exercise in patients with CKD stage G4 on the aforementioned hard end points. Third, although the subjects were randomized into both groups, the parameters during the pre-exercise period were obtained retrospectively, and the eGFR slope of the pre-exercise period tended to be steeper in the exercise group than in the control group. Furthermore, the period between each visit depended on the medical condition and discretion of the attending physician, especially during the pre- and postexercise periods, and variation was observed in the number of eGFR measurements, which could affect the eGFR slope calculation. Finally, it was difficult to evaluate whether aerobic exercise, resistance exercise, or both types of exercise contributed to the results. Additional trials with a head-to-head comparison of aerobic exercise and resistance exercise are needed to answer this question.

In conclusion, participants randomly assigned to the exercise group exhibited significant improvement in eGFR from the pre-exercise period to the midexercise period, although the improvement in eGFR slope was attenuated from the pre-exercise period to the postexercise period.

## Disclosure

All the authors declared no competing interests.

## Supplementary Material

**Supplementary Methods.**

**Supplementary References.**

**Table S1.** Longitudinal data during the study period of the study groups.

**Figure S1.** Flowchart of the study process.

**Figure S2.** CONSORT diagram of the flow of patients throughout the various phases of the trial.

**Figure S3.** Estimated glomerular filtration rate (eGFR) slope during the pre-exercise, midexercise, and postexercise periods, including all analyzed participants (*n* = 23 for each group).
