# Supplementary material for: Home-Based Exercise Program Ameliorates Renal Function Decline in Patients With CKD Stage 4
Source: Kidney Int Rep. 2022 Jan 7;7(4):899–903. doi: 10.1016/j.ekir.2022.01.006 (PMC9039468; doi:10.1016/j.ekir.2022.01.006)

Methods

Study population

In total, 46 outpatients with stage 4 chronic kidney disease (CKD) aged 20–90 years with the ability to understand and provide informed consent were recruited and randomly allocated into the exercise (23 patients) or control group (23 patients) in a previous randomized controlled trial (RCT) conducted between November 2018 and June 2020 at Keio University Hospital, Tokyo, Japan (approval number: 20180125; UMIN-CTR number: UMIN0000034855).[S1] The protocol of this post-hoc analysis was independently reviewed and approved by the ethics committee (approval number: 20200342). This study adhered to Consolidated Standards of Reporting Trials and the Declaration of Helsinki.

Estimated glomerular filtration rate (eGFR) was calculated from serum creatinine using three-variable Japanese equations.[S2] Patients whose eGFR slopes before and after the intervention could not be calculated due to insufficient data were excluded. Consequently, none of the patients were excluded, and all eligible patients agreed to participate. The effect size of the exercise intervention on renal function was calculated as 0.95 based on the RCT on patients with predialysis CKD with an eGFR of 20–60 mL/min/1.73 m^2.[S3] Moreover, 38 patients were required to achieve a power of 0.80 using α = 0.05 in the analysis of covariance. Additionally, the statistical power was calculated as 0.88 with 46 participants.

Outcome measures

The primary outcome was the effect of the exercise intervention on the decline in renal function from 48 weeks before the start of the intervention (pre-exercise), during the intervention (24 weeks, mid-exercise), and 24 weeks after the end of intervention (post-exercise) (Supplementary Figure S1). We assessed the eGFR slope (mL/min/1.73 m^2/year) to define the renal function decline, calculated using the least squares method for secular change in each period. Accordingly, all eGFR measurements at the outpatient clinic from 48 weeks prior to the intervention to the start of the intervention (eGFR at the start of the intervention was included), from the start of the intervention to the end of the 24-week intervention (eGFR at the start and end of the 24-week intervention were included), and from the end of the 24-week intervention to 24 weeks after the end of the intervention (eGFR at the end of 24-week intervention was included) were used to calculate the eGFR slope during the pre-exercise, mid-exercise,

and post-exercise periods, respectively. Secondary outcomes included changes in body weight (BW; kg) with body mass index (BMI; kg/m^2), blood pressure (BP) (including systolic BP, diastolic BP, and mean BP), and heart rate (HR) in each period over time between the study groups. All outcome measures, including anthropometric data, biochemical analyses, and physical functioning were evaluated upon study initiation and at the end of the 24-week intervention in all available patients, as previously described.[S1] Given that eGFR, BW, BP, and HR were measured in every CKD outpatient clinic, values for each outpatient clinic were retrospectively obtained from medical records available from the pre- to post-exercise period.

Exercise intervention

During the intervention period, patients in the exercise group were instructed to perform unsupervised home-based individualized aerobic exercise (AE) thrice weekly and resistance exercise (RE) twice weekly for 24 weeks, as previously described.[S1, S4] Briefly, the AE training target was 40%–60% of the peak HR, as determined at baseline with the ISWT, with 11–13 on the Borg rate of perceived exertion scale. Moreover, the RE training target was 70% of their one-repetition maximum. The exercise capacity of the participants was reassessed bimonthly during CKD clinic visits, after which the exercise prescription was adjusted accordingly. Additionally, only patients in the exercise group were sent two postcards every other week to determine whether they performed AE and/or RE each day. Consequently, among the patients in the exercise group who underwent the exercise capacity assessment, collection rate of postcards and adherence to prescribed AE, RE, and AE + RE sessions were calculated as 100% (96–100), 92% (40–99), 96% (85–100), and 87% (34–96), respectively. [S1] The control group received usual care and was asked to maintain their lifestyle during the period. The physical activity of the control group was not monitored. At the start of the intervention, physical activity was assessed using the short version of the International Physical Activity Questionnaire in both groups.[S5, S6]

At the end of the 24-week intervention period, all participants received instruction to perform home-based individualized AE and RE, although reassessment of exercise capacity, adjustment of exercise prescription, and monitoring of adherence to the exercise program via postcard were not performed.

Statistical analysis

The normality of continuous data was tested using the Kolmogorov–Smirnov test. Normally and non-normally distributed data were described as mean ± standard deviation and median (interquartile range), respectively, whereas categorical data were expressed as *n* (%).

Unpaired Student's t-test or the Mann–Whitney U test for continuous variables and Fisher's exact test for categorical variables were used to compare the baseline characteristics between groups. The effects of the study group over time were assessed using linear mixed models to determine the effectiveness of the intervention on outcomes,[S7, S8] which included the following fixed effects: group, time (pre-exercise, mid-exercise, and post-exercise), age, and sex. Additionally, the models were adjusted for the baseline value of each dependent variable to increase the precision of the estimates. Furthermore, an interaction term was included as a fixed effect, time × group, which indicated the effect of the study group over time. SPSS software for Mac (ver. 27; IBM Corp., NY, USA) was used to perform all statistical analyses, with $P < 0.05$ indicating statistical significance.

References

S1. Uchiyama K, Adachi K, Muraoka K, et al. Home-based aerobic exercise and resistance training for severe chronic kidney disease: a randomized controlled trial. *J Cachexia Sarcopenia Muscle*. 2021. DOI: 10.1002/jcsm.12775.

S2. Matsuo S, Imai E, Horio M, et al. Revised equations for estimated GFR from serum creatinine in Japan. *Am J Kidney Dis*. 2009;53:982–992.

S3. Greenwood SA, Koufaki P, Mercer TH, et al. Effect of exercise training on estimated GFR, vascular health, and cardiorespiratory fitness in patients with CKD: a pilot randomized controlled trial. *Am J Kidney Dis*. 2015;65:425–434.

S4. Uchiyama K, Washida N, Morimoto K et al.. Home-based aerobic exercise and resistance training in peritoneal dialysis patients: a randomized controlled trial. *Sci Rep*. 2019;9:2632.

S5. Craig CL, Marshall AL, Sjöström M, et al. International physical activity questionnaire: 12-country reliability and validity. *Med Sci Sports Exerc*. 2003;35:1381–1395.

S6. Hamada M, Yasuda Y, Kato S, et al. The effectiveness and safety of modest exercise in Japanese patients with chronic kidney disease: a single-armed interventional study. *Clin Exp Nephrol.* 2016;20:204–211.
S7. Meuleman Y, Hoekstra T, Dekker FW, et al. Sodium restriction in patients with CKD: A randomized controlled trial of self-management support. *Am J Kidney Dis*. 2017;69:576–586.
S8. Humalda JK, Klaassen G, de Vries H, et al. A self-management approach for dietary sodium restriction in patients with CKD: A randomized controlled trial. *Am J Kidney Dis*. 2020;75:847–856.

Supplementary Table S1. Longitudinal data during the study period of the study groups

	48 weeks before the intervention			At the start of the intervention			At the end of the intervention			24 weeks after the end of the intervention		
	**Control**	**Exercise**	***P*-value**	**Control**	**Exercise**	***P*-value**	**Control**	**Exercise**	***P*-value**	**Control**	**Exercise**	***P*-value**
eGFR (ml/min/1.73 m^2)	25.2 ± 4.7	26.1 ± 4.7	0.53	23.8 ± 4.5	22.4 ± 5.1	0.32	22.0 ± 4.3	21.3 ± 6.7	0.68	20.9 ± 5.2	20.5 ± 8.5	0.85
Body weight (kg)	59.7 ± 13.4	66.3 ± 15.5	0.13	60.3 ± 12.9	66.6 ± 15.9	0.15	61.3 ± 12.9	66.6 ± 16.3	0.23	60.9 ± 13.1	66.0 ± 15.8	0.24
Body mass index (kg/m^2)	22.8 ± 4.7	24.6 ± 4.4	0.2	23.0 ± 4.3	24.7 ± 4.6	0.23	23.4 ± 4.4	24.7 ± 4.8	0.33	23.3 ± 4.5	24.5 ± 4.7	0.37
Systolic blood pressure (mmHg)	138.2 ± 17.9	139.3 ± 15.5	0.83	140.8 ± 15.5	140.2 ± 16.3	0.9	144.0 ± 19.9	142.3 ± 18.7	0.76	149.3 ± 15.7	140.2 ± 18.3	0.08
Diastolic blood pressure (mmHg)	74.5 ± 17.0	78.8 ± 13.1	0.34	75.7 ± 12.2	78.5 ± 14.4	0.48	76.9 ± 12.7	78.5 ± 16.3	0.72	76.7 ± 12.9	75.7 ± 16.0	0.82
Mean blood pressure (mmHg)	95.7 ± 15.7	99.0 ± 12.7	0.45	97.4 ± 12.1	99.0 ± 13.8	0.67	99.3 ± 13.0	99.3 ± 13.0	0.92	100.9 ± 11.7	97.2 ± 15.7	0.37
Heart rate (bpm)	71.8 ± 15.4	76.3 ± 18.3	0.37	76.2 ± 16.6	80.0 ± 16.8	0.44	73.9 ± 13.8	72.7 ± 14.0	0.76	75.0 ± 12.5	77.5 ± 14.0	0.54
Use of antihypertensive agents/diuretics												
CCBs	13 (56%)	16 (70%)	0.54	12 (52%)	15 (65%)	0.55	12 (52%)	16 (70%)	0.37	13 (56%)	15 (65%)	0.76
RAS inhibitors	12 (52%)	15 (65%)	0.55	11 (48%)	15 (65%)	0.37	9 (39%)	15 (65%)	0.14	8 (35%)	13 (57%)	0.24
BBs	2 (9%)	5 (22%)	0.41	2 (9%)	5 (22%)	0.41	2 (9%)	5 (22%)	0.41	2 (9%)	5 (22%)	0.41

Loop diuretics	5 (22%)	3 (13%)	0.7	3 (13%)	4 (17%)	1	3 (13%)	4 (17%)	1	3 (13%)	5 (22%)	0.7
Thiazide diuretics	2 (9%)	1 (4%)	1	2 (9%)	2 (9%)	1	1 (4%)	1 (4%)	1	1 (4%)	1 (4%)	1
Others	6 (26%)	2 (9%)	0.24	4 (17%)	3 (13%)	1	4 (17%)	3 (13%)	1	5 (22%)	2 (9%)	0.41

Abbreviations: eGFR, estimated glomerular filtration rate; CCBs, calcium channel blockers; RAS, renin–angiotensin system; BBs, β blockers.

Supplementary Figure S1. The flow chart of the study process

[*] Usual care without exercise instruction

[†] Although all patients in both the control and exercise groups received instruction to perform home-based individualized exercise at 24 weeks (at the end of the intervention), reassessment of exercise capacity, adjustment of the exercise prescription, and monitoring of adherence to the exercise program by postcard were not performed.

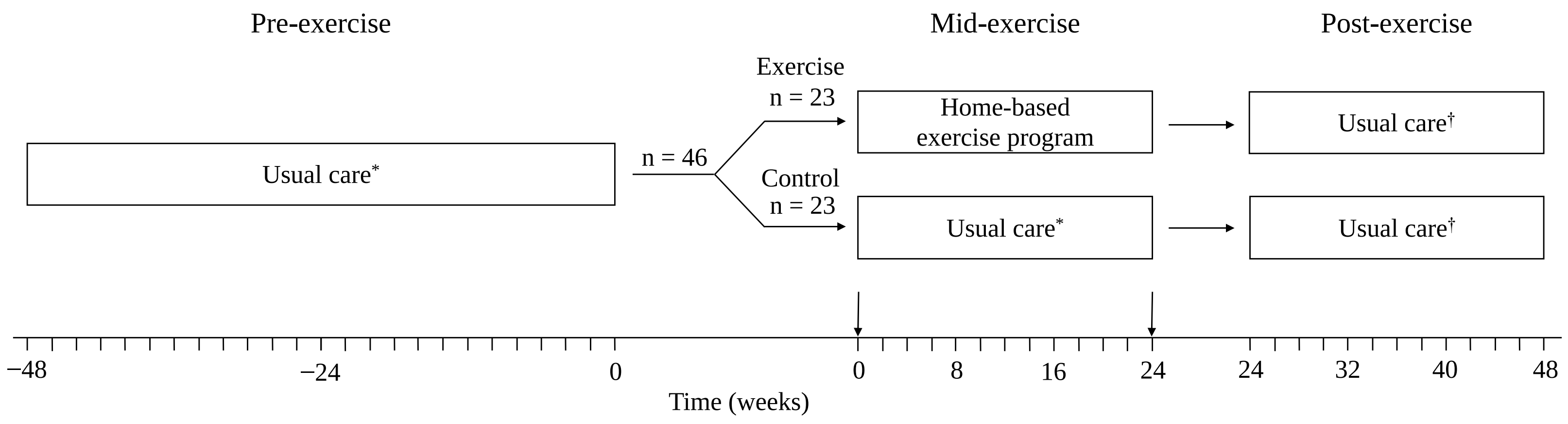

Supplementary Figure S2. CONSORT diagram of the flow of patients through the various phases of the trial

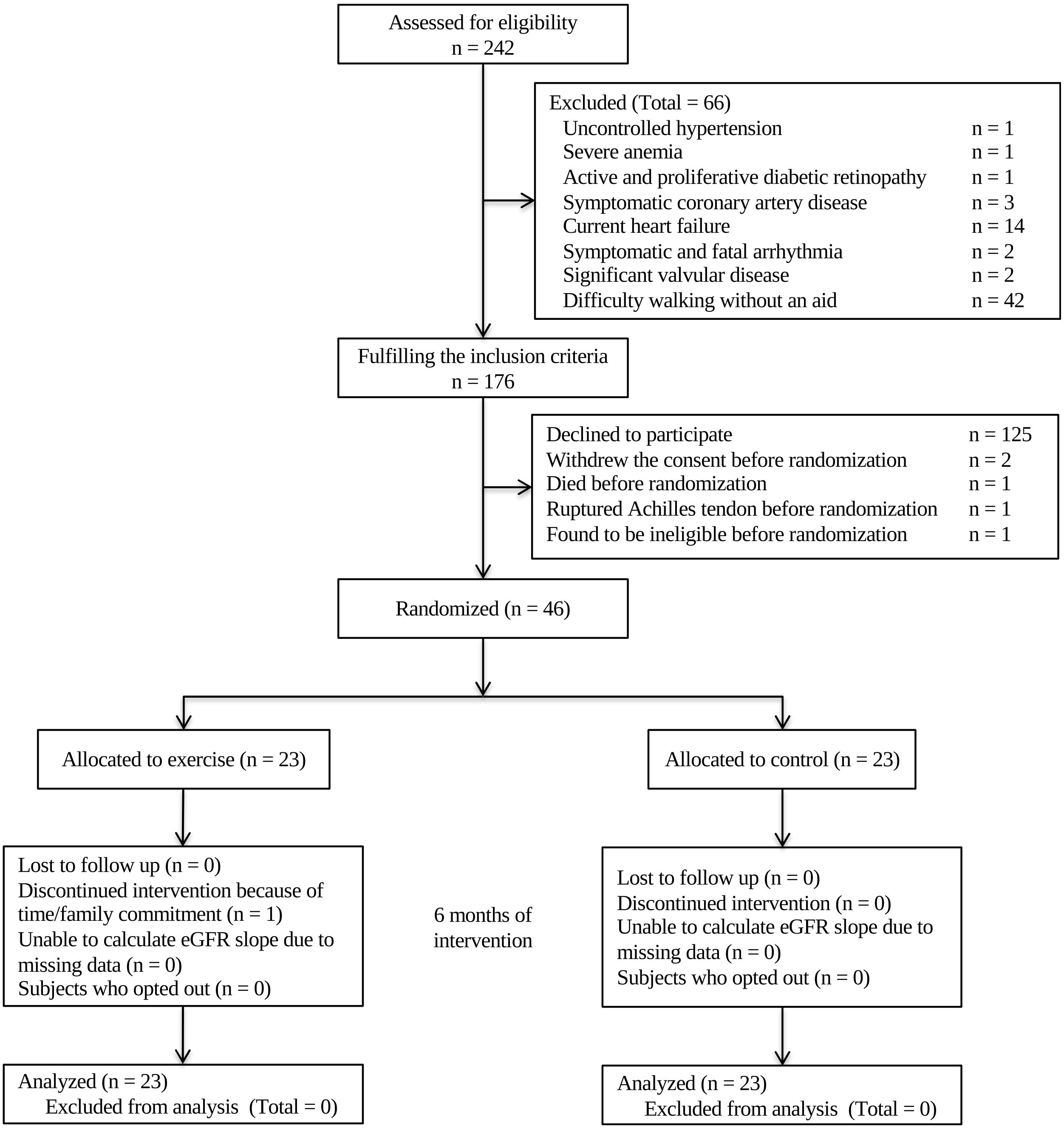

Supplementary Figure S3. Estimated glomerular filtration rate (eGFR) slope during the pre-exercise, mid-exercise, and post-exercise periods including all analyzed participants (n = 23 for each group). * *P*-value = 0.02 versus the control group.

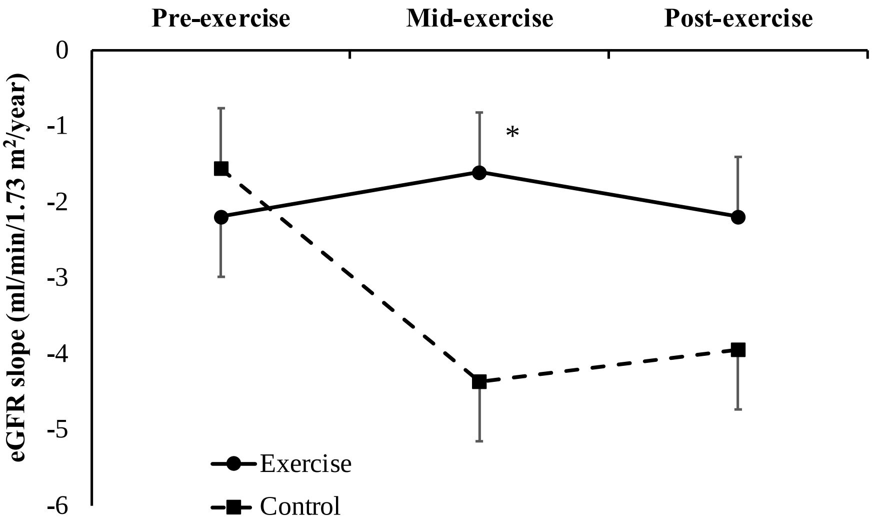

Supplement: Supplementary File (PDF) [file mmc1.pdf]
